# Insight into the Use of Brewers’ Spent Grain as a Low-Carbon Aggregate in Building Materials

**DOI:** 10.3390/biomimetics9120781

**Published:** 2024-12-21

**Authors:** Badreddine El Haddaji, Mohammed-Hichem Benzaama, Marc Quiertant, Yassine El Mendili

**Affiliations:** 1 Institut de Recherche ESTP, 94230 Cachan, France; belhaddaji@estp.fr (B.E.H.); hbenzaama@estp.fr (M.-H.B.); yelmendili@estp.fr (Y.E.M.); 2 ESITC Paris, 94110 Arcueil, France

**Keywords:** brewers’ spent grains, gelatinization of starch, bio-organic binders, thermal conductivity, sustainable materials, thermal insulation, hygrothermal properties, mechanical characterization

## Abstract

This study investigates the use of Brewers’ Spent Grains (BSGs) as a sustainable biocomposite building materials, using cornstarch as a biopolymer binder. BSG aggregates are compared with hemp shives, a conventional aggregate known for its thermal properties. Starch is employed as a natural binder in three different formulations to further reduce the carbon footprint of the building material. Considering aggregates, the first formulation contains only BSGs, the second consists of half BSGs and half hemp shives, and the third uses only hemp shives. In addition, morphological analysis using Scanning Electron Microscopy (SEM) is conducted to examine the microstructure and porosity of the raw BSG and hemp shives. Hygrothermal properties are measured using Heat Flow Meter (HFM) and Dynamic Vapor Sorption (DVS) techniques, while mechanical properties are also assessed. Results indicate that the thermal conductivity of the BSG formulation (0.131 W/(m·K)) is double that of the hemp shives formulation (0.067 W/(m·K)), whereas the mixed BSG/hemp shives formulation exhibits a thermal conductivity of 0.106 W/(m·K). However, DVS measurements reveal better hygrothermal properties for the BSG formulation compared to the hemp shives formulation. Lastly, mechanical properties are found to be nearly equivalent across the three formulations. These findings suggest that BSG waste has potential as a viable material for use in construction. Further work on formulation optimization and durability is necessary to fully realize the potential of this waste in promoting a circular economy within the building materials industry.

## 1. Introduction

As global environmental concerns intensify, the search for sustainable materials has become a key priority across industries. This is particularly the case in the construction sector, one of the largest contributors to carbon emissions [1,2,3]. Studies have shown that material emissions contribute a substantial portion of total emissions in buildings, ranging from 32 to 48% [4]. Embodied carbon emissions from construction materials have been quantified in various studies, with steel and concrete identified as the most carbon-emitting materials [5]. Strategies to reduce carbon emissions in buildings include using local and reused materials, which have been shown to have a significant impact on reducing embodied carbon emissions [5]. Among these strategies for reducing carbon emissions is the reuse and transformation of local waste from across the spectrum of human activities into unexpected sources of low-carbon alternatives for building materials. Multiple studies in the literature use this strategy with wastes from different origins. Example of such wastes includes waste sludges, demolition waste, plastic waste, and seashells wastes, which show the potential of some waste in building materials [6,7,8,9,10]. Properties of building materials containing such waste remain as good as those of conventional material for multiple applications [11,12].

**Figure 1:**
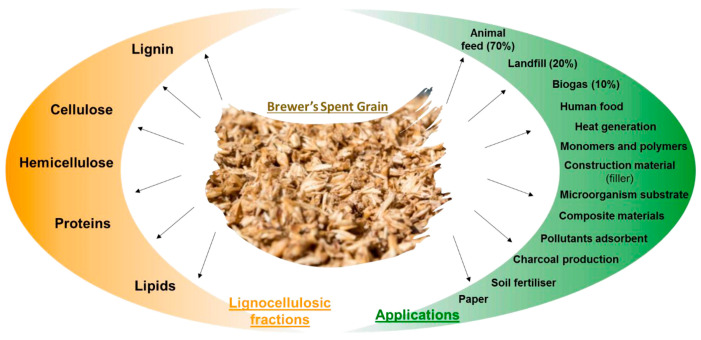
Fractions present in BSGs’ lignocellulosic biomass and their potential applications [14].

Brewers’ Spent Grain (BSG) is a by-product of the brewing process that has the potential for valorization in various industries, including construction [13]. Most BSGs end up in animal feed, in landfills, and as biogas, with lesser volumes being used for polymers, soil fertilizer, and paper, as shown in Figure 1. These applications are possible thanks to the fractions that can be found in BSGs, such as lignin, cellulose, hemicellulose, proteins, and lipids [14]. In the EU alone, there is an estimated 10.02 million tons of BSGs generated each year (2020) [15]. The current fetch price of this waste is EUR 40 a wet ton, and it can reach, for example, a market price of around EUR 180 a ton after being processed for bio-refinery applications [16]. However, storage conditions are important due to the microbiological activity of BSGs, as it can lead to fungal development [17]. This can be detrimental for the final application of this product and should be taken into consideration.

The use of spent grains in building materials is a topic that has gained attention in recent years. Brewers’ Spent Grains have been used in the production of lignin, a structural component with efficient thermal properties [18]. One study found that spent grains could be used to increase porosity in bricks, making them suitable for building materials [19]. Additionally, spent grains have been investigated for their potential in thermally insulating cellulose nanofiber aerogels, highlighting their versatility in different applications [20]. Furthermore, the valorization of Brewer’s Spent Grain has been explored to produce biopolymer composite materials and bio-based building blocks [14]. BSG has emerged through the literature as a promising alternative to traditional building material. Indeed, this by-product of the brewing industry holds potential to contribute to sustainable construction practices.

The components of BSG (Figure 1) are used in building materials. These components are commonly known as lignocellulosic biomass, derived from agricultural waste, and are composed of various constituents, such as cellulose, hemicellulose, lignin, pectin, and protein [21]. These components have been explored for their potential use in building materials for insulation purposes. Cellulose, hemicellulose, and lignin have been identified as key components in this regard [22]. Furthermore, the antimicrobial properties of these natural biopolymers have been investigated, showcasing their potential as effective agents in building materials [23]. Additionally, the production of algal cellulose has been explored, emphasizing its potential use in building materials due to its composition of lipids, carbohydrates, and proteins [24]. In search of sustainable building materials, the use of biodegradable polymers has gained traction to reduce non-biodegradable plastic waste [25]. Bioplastics derived from cellulose and hemicellulose have also been studied for their potential applications in building materials [26]. Hence, common sense indicates that introducing BSGs into building materials is not a far-fetched idea and can bring benefits to the building materials industry with an additional purpose of reusing widely available waste in some regions.

In this work, we investigate the potential of using BSG as an aggregate in building materials. First, preconditioning of the raw BSG is studied and an adequate protocol is used to hamper the fungal development and ensure accurate dosing when formulating. Then, we choose to use raw BSG materials as a material for isolation that could increase energy efficiency of buildings. To achieve this goal, we have compared several formulations using cornstarch as a low carbon binder. We have chosen hemp shives as a commonly studied reference aggregate for comparison. Therefore, three formulations are studied, the first one with only BSG as aggregates, the second formulation with half hemp shives and half BSG and the third formulation with only hemp shives. We then have characterized the physical, mechanical and thermal properties of the studied formulations for potential use in insulations panels.

## 2. Materials and Methods

### 2.1. Materials

#### 2.1.1. Brewers’ Spent Grain

Brewers’ Spent Grain (BSG) is a by-product of the beer-brewing process, consisting primarily of leftover husks, seeds, and other solid materials from malted barley. This material is typically rich in fibers, proteins, and other organic components. Sourced from local breweries, BSG is collected after the brewing process, where it would otherwise go to waste or be used for limited purposes such as animal feed.

In this study, we focus on repurposing BSG to produce sustainable insulation panels. The fibrous nature of BSG makes it a promising candidate for use as a bio-based aggregate in building materials, particularly in panels designed for thermal insulation. By using BSG, we aim to reduce the reliance on conventional, carbon-intensive materials, while supporting circular economic principles and promoting the local sourcing of raw materials from breweries.

The composition of BSG from literature ranges from 19.2% to 40% for hemicellulose, 12% to 33% for cellulose, 1% to 2.7% for starch, 14.2% to 26.7% for protein, 11.5% to 22% for lignin, 3% to 13% for lipids, 1.1% to 4.6% for Ash and 0.7% to 2% for phenolics [13].

**Figure 2:**
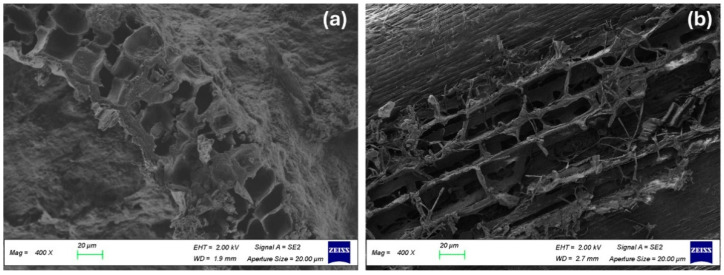
SEM images of (**a**) Brewers’ Spent Grain and (**b**) hemp shives.

The SEM image of BSG in Figure 2a shows a relatively dense surface with some visible micropores scattered across the structure. These pores are primarily formed by the fibrous remnants of the grain husks, which contribute to its moderate porosity. However, the compactness of the BSG structure limits the overall pore volume.

#### 2.1.2. Hemp Shives

Hemp shives, the woody core of the hemp plant, are sourced from local farmers in Bar-sur-Aube, situated in the Champagne-Ardenne region of France. These shives are a by-product of hemp cultivation, primarily used for fiber production, and consist of lightweight, fibrous material with excellent thermal and acoustic properties. The thermal conductivity of hemp shives is 0.05 W/(m·K).

In this project, hemp shives are repurposed for the production of sustainable insulation panels. Their natural insulating capacity, coupled with their local availability, makes them an ideal bio-based aggregate for environmentally friendly building materials. By using hemp shives, we aim to reduce the environmental impact of insulation materials, support local agriculture, and promote the use of renewable, low-carbon resources in construction.

The composition of hemp shives from the literature ranges from 44% to 51.6% for cellulose, from 6.4% to 27% for hemicellulose, from 8.1% to 28% for lignin, from 1.0% to 29.4% for soluble compounds, from 0.6% to 8.8% for proteins, and from 1.0% to 6.6% for ashes [27]. The used hemp shives were provided from ISOCANNA^®^ Saint-Astier in Saint-Astier, France.

In contrast to what was observed for BSGs, the SEM image of hemp shives in Figure 2b reveals a significantly more porous structure, with large, well-defined macropores. The fibrous and woody nature of hemp shives creates a highly open and irregular texture, allowing for greater pore connectivity and a higher overall porosity compared to BSGs. This increased porosity is advantageous for applications requiring enhanced insulation and moisture regulation.

Table 1 provides a summary of the physical properties of Brewers’ Spent Grains and hemp shives.

#### 2.1.3. Binder

Table 2 presents the chemical composition of cornstarch. The analysis of its composition shows that cornstarch is distinguished by a relatively high concentration of polysaccharides, specifically amylopectin and amylose, while the levels of extractives, including crude fats, proteins, and ash, are notably low.

The amylose content, which is the primary component of native starches, was measured at 24.6 g per 100 g of starch. This concentration falls within the typical range of amylose content for native plant starches, which is between 14% and 29%.

Cornstarch, primarily composed of amylose and amylopectin, undergoes gelatinization when heated in water. At 80 °C, the starch granules absorb water, causing them to swell and break the hydrogen bonds that hold the glucose molecules together. This swelling disrupts the crystalline structure of amylopectin, leading to a loss of birefringence. As the temperature rises, some of the amylose is released into the surrounding solution, while amylopectin remains in the swollen granules, increasing the viscosity of the mixture. The interaction of amylose and amylopectin with water forms a viscoelastic network, which traps water and gives the material its thickening and stabilizing properties. Upon cooling, amylose and amylopectin chains may partially recrystallize, a process known as retrogradation, which can strengthen the structure of the gel but may also cause syneresis, or water release.

In this study, we used gelatinized cornstarch as a bio-organic binder to formulate the targeted sustainable building materials. Corn-derived gelatinized starch, processed at 80 °C, forms a natural adhesive that promotes particle cohesion in plant fiber-based composites or biosourced aggregates. Acting as a natural polymer, it creates a viscoelastic network that improves the mechanical stability and moisture retention of the material. The use of cornstarch as a binder contributes to reducing the carbon footprint of building materials, aligning with circular economic principles, and providing an eco-friendly alternative to synthetic binders while maintaining comparable mechanical performance.

#### 2.1.4. Water

In this study, gelatinized starch was prepared using local tap water. The elemental composition of the tap water used for mixing is presented in Table 3. The pH level of the tap water was recorded at 7.73. Understanding the composition of the water, particularly its pH, is essential, as it can influence the characteristics of the material. Moreover, this information is crucial for replicating and comprehending the experimental conditions.

### 2.2. Methods

#### 2.2.1. Sample Preparation

As already mentioned, we have developed three distinct formulations for biocomposite building materials using BSGs and hemp shives as aggregates, with gelatinized starch serving as the binder:

- Formulation 1 (BSG100): This formulation contains only BSGs.
- Formulation 2 (BSG50): This formulation consists of equal parts (50% each) of BSGs and hemp shives.
- Formulation 3 (BSG0): This formulation uses only hemp shives.

To cast the biocomposite materials, we prepared two types of samples: cubic samples for hygroscopic and thermal testing, and cylindrical samples for compressive strength testing. The dimensions and specifications for the samples are as follows:

- Cylindrical samples: These were produced in disposable molds with dimensions of 11 cm in diameter and 22 cm in height. The cylindrical geometry was chosen to facilitate mechanical testing, particularly compressive strength, which benefits from consistent load distribution across a circular cross-section.
- Cubic samples: These were produced using reusable wooden molds with dimensions of 25 cm in height, 25 cm in length, and 7 cm in thickness. The cubic shape was selected to optimize thermal property measurements, such as thermal conductivity, as the larger flat surface area ensures accurate placement of sensors and uniform heat flow during testing.

In typical applications for starch-based insulating panels, the starch content in the mixture is set at 12.5% of the total mass of the panel.

**Figure 3:**
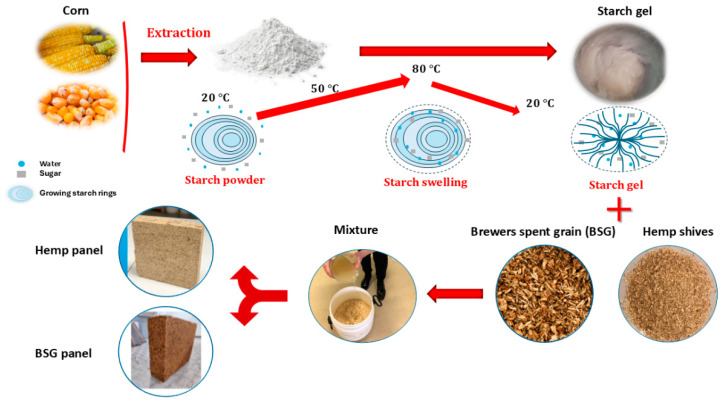
Overview of the manufacturing process for biocomposite building materials using Brewers’ Spent Grains and hemp shives, with gelatinized starch as a binder.

To cast the biocomposite materials (Figure 3), we began by heating water to 80 °C for the gelatinization of starch. Once the desired temperature was reached, we gradually added starch to the water while stirring continuously until a homogeneous paste was obtained; this paste served as the binder for our material. While the starch was gelatinized, we prepared the BSGs in a mixer. Once the starch paste was ready, we incorporated it into the prepared BSGs in the mixer. We then mixed the combined ingredients for approximately 10 min, pausing for 5 min during this period to homogenize the mixture, ensuring that any non-impregnated fibers located on the periphery of the mixer were fully integrated. This step is crucial for achieving a homogeneous blend that optimally uses the properties of the individual components. After obtaining a consistent mixture, we poured the material into the prepared cylindrical and cubic molds. Finally, once the samples were molded, we stored them in an oven at 23 °C and 50% humidity for 28 days. After this curing period, the characterization test could begin.

By following this procedure, we can create effective biocomposite materials with the potential for use in sustainable building applications, leveraging the natural properties of BSG and hemp shives, along with the binding capacity of gelatinized starch.

#### 2.2.2. Characterization Techniques

The compressive tests were performed in accordance with EN-12390-3 on 11–22 cm cylindrical specimens. After the specified curing period, each specimen was removed from the curing environment and measured to confirm that the diameter was 11 cm and the height was 22 cm. The compressive strength of samples was evaluated using an IGM testing equipment with a 50 kN capacity. The load was applied at a controlled rate of 1 mm/min until the specimen failed. Results were reported in megapascals (MPa).

Heat Flux Meter (HFM) measurements were conducted using a (Netzsch HFM 446, Selb, Germany) to evaluate the thermal transmittance of the building material. The panel samples, with dimensions of 25 × 25 × 7 cm^3^, were placed in a testing chamber with controlled temperature conditions. The HFM sensors were affixed to the surface of the material to measure the rate of heat flow through it. Temperature sensors were installed on both sides of the panel to record the internal and external surface temperatures. The system was maintained at a steady-state temperature gradient, and data on heat flux and temperature differences were continuously recorded. The thermal transmittance (U-value) was computed based on the measured heat flux and temperature differential, providing an assessment of the material’s insulation performance.

Dynamic Vapor Sorption (DVS) measurements were performed using a Vsorp Moisture Sorption Analyzer apparatus supplied from (Mercer Instruments, Passy, France) to assess the moisture sorption characteristics of the BSG samples. Small samples were placed in the chamber, where they were exposed to controlled humidity levels ranging from 0% to 95% relative humidity (RH) in incremental steps. The weight changes of the sample were continuously monitored as it absorbed or desorbed moisture. Equilibrium was assumed once the sample weight stabilized at each RH step. The data were used to generate sorption isotherms, providing insights into the material’s moisture sensitivity and capacity.

The chemical composition of the tap water was analyzed using a (Thermo Scientific DIONEX ICS3000 CC, Villebon-sur-Yvette, France) ion chromatography system. This system is equipped with a dual-pump module, a chromatography detector module, an eluent generator module, and an autosampler. The column pressure and eluent flow rate were set to 1.0 mL/min. Ionic species were identified and quantified by interpolating against a suitable calibration curve, with all measurements performed at room temperature.

The fibers and Brewers’ Spent Grains (BSGs) were analyzed to determine their specific surface area and absolute density. The specific surface area was measured using the Brunauer–Emmett–Teller (BET) method, which relies on nitrogen gas adsorption [28]. Samples were dried at 50 °C for 24 h to eliminate moisture before undergoing adsorption in a BET apparatus. Absolute density was measured using a helium pycnometer (Accupyc II 1340, Microméritics, Mérignac, France), where dried samples were placed in a chamber with helium gas to determine their true volume by gas displacement, and density was calculated as the ratio of mass to volume.

Morphological characterization was conducted using a scanning electron microscope (SEM; SUPRA 55 SAPPHIRE; Carl Zeiss, Jena, Germany). BSG and hemp shives were cleaned with ethanol and oven-dried at 50 °C for 24 h. They were embedded in an epoxy resin mixed with hardener, poured into molds, and cured as per the resin’s specifications. The cured sample was polished to expose the samples and create a smooth surface (<0.3 µm). To ensure conductivity, a thin silver coating was applied via sputtering. The prepared sample was mounted on a conductive SEM stub using carbon adhesive, ensuring proper charge dissipation for SEM imaging at 20 kV.

To assess the apparent porosity of the panel samples, the gravimetric technique outlined in ISO 5017 was employed. Initially, the samples were saturated with water and weighed while suspended on a scale. They were then submerged in water ($M_{w}$) before being weighed again in the air ($M_{o}$). After drying in an oven, the dry weight ($M_{d}$) was recorded. The apparent porosity was subsequently computed using the following Equation (1):

(1) $$\rho_{d}=\rho_{w}*\frac{M_{d}}{M_{o}-M_{w}}$$

where $\rho_{d}$ is the apparent density of the panel sample.

## 3. Results

### 3.1. Moisture Sorption Isotherm of Panel Specimens

**Figure 4:**
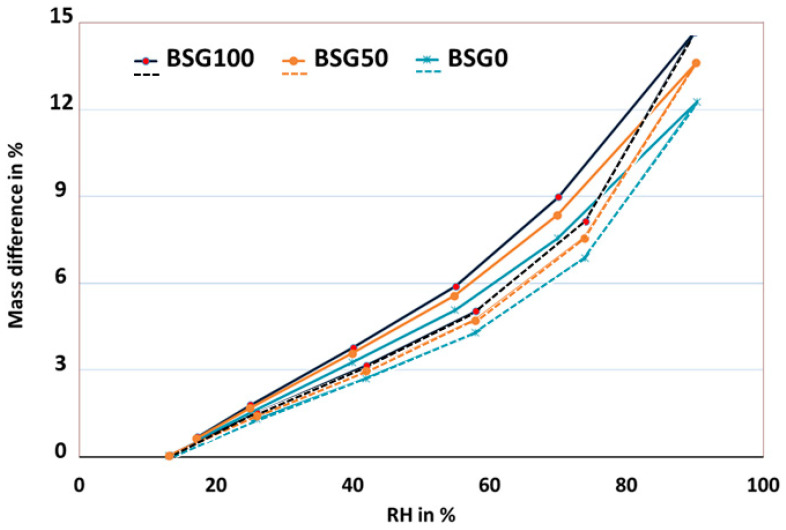
Moisture sorption isotherms for the studied formulations at room temperature. Continuous line: adsorption. Dashed line: desorption.

Figure 4 illustrates the typical mass change during adsorption and desorption processes at various RH levels. In this figure, it can be seen that the moisture sorption curves for all samples exhibited a sigmoidal shape, indicating Type II isotherm behavior according to the IUPAC classification. This isotherm type is characteristic of porous materials, such as the formulations investigated in this study.

The mass differential values increased from BSG0 to BSG50, reflecting the influence of porosity on this phenomenon. Notably, BSG100, despite having the highest density and lowest porosity, exhibits a significant capacity for moisture absorption due to the hydrophilic nature of the BSG. In contrast, BSG0, which consists solely of hemp shives, demonstrates a different moisture absorption profile; while it has higher porosity, its ability to retain moisture is lower compared to BSG100. BSG50, with equal parts of both materials, shows intermediate absorption characteristics.

The comparison between hemp shives and BSGs reveals that while hemp shives contribute to the overall porosity and can enhance moisture retention, BSGs have a stronger capacity to absorb moisture due to their composition. This high absorption capacity of BSG100 can inhibit surface condensation by promoting uniform moisture distribution throughout the formulation structure.

**Figure 5:**
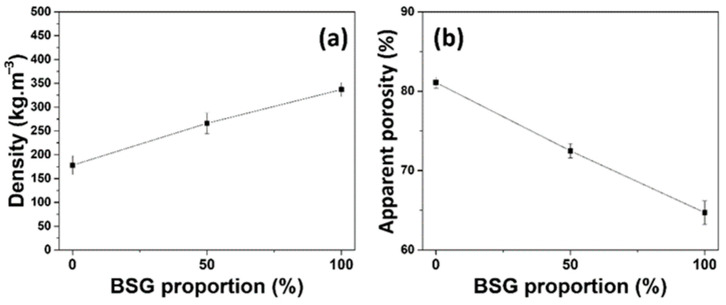
(**a**) Density of specimens with different proportions of BSG and (**b**) apparent porosity of panels with different proportions of BSG.

Figure 5a shows the decrease in density from approximately 350 kg/m^3^ with 100% BSGs to 175 kg/m^3^ with 100% hemp shives; this decrease can be explained by the inherent differences in the bulk densities of the raw materials, as detailed in Table 1. Specifically, raw BSGs have a bulk density nearly six times greater than that of hemp shives. This difference results in lower mass per unit volume for formulations containing hemp shives, leading to reduced overall density. Additionally, the observed increase in porosity, from 65% with 100% BSGs to 81% with 100% hemp shives, is influenced by the structural characteristics of the materials. Hemp shives, being less compact and more fibrous, naturally introduce larger void spaces within the mixture, enhancing porosity. The hydrophilic nature of both materials further contributes to this behavior by facilitating water absorption and swelling during sample preparation, thus increasing the volume without substantially affecting the mass. Furthermore, the compaction process during panel formation may align the particles in a manner that accentuates void creation, particularly in samples with a higher hemp shive content. This phenomenon is most pronounced in BSG0, where the combination of lower material density and increased porosity aligns with the properties of the constituent aggregates.

### 3.2. Thermal Conductivity of Panel Specimens

**Figure 6:**
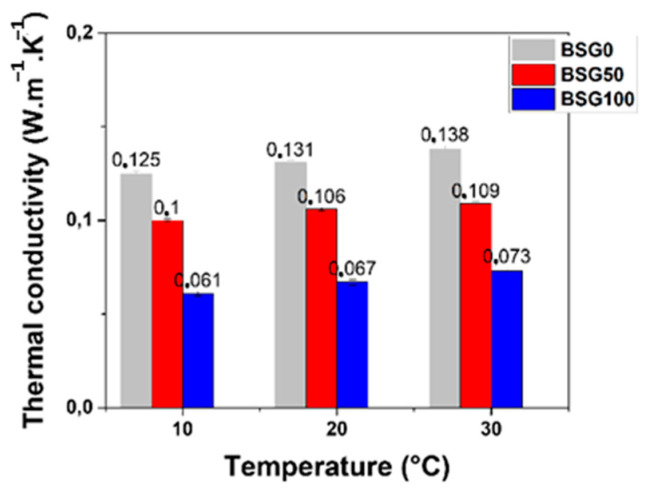
Thermal conductivity vs. temperature of specimens with different proportions of BSGs.

As shown in Figure 6, the thermal conductivity of the panels formulated solely with BSGs was measured to be 0.131 W/(m·K) at 20 °C, which is significantly higher than that of the formulation containing only hemp shives, as the latter exhibits a thermal conductivity of 0.067 W/(m·K). The mixed formulation (BSG50) shows an intermediate thermal conductivity of 0.106 W/(m·K).

These results indicate that the incorporation of BSGs into the panel significantly influences thermal conductivity. The lower thermal conductivity of the hemp shive formulation suggests its superior insulating properties, while the mixed formulation reflects a balance between the two materials’ thermal characteristics.

The insulating potential of the panels can be further enhanced through increased porosity within the material. The incremental substitution of hemp shives with BSGs shows that organic additives like BSGs contribute to pore development based on the physical properties of the organic additives, leading to improved insulation performance and mechanical properties. Additionally, the higher specific heat capacity of the BSG formulation plays a crucial role in reducing thermal gradients, which is essential for maintaining comfortable indoor temperatures.

Incorporating BSGs in panel construction improves thermal characteristics, minimizing heat transfer and enhancing thermal comfort for occupants. The thermal insulation capabilities of both BSGs and hemp shives are noteworthy. This relationship highlights the significance of energy efficiency and environmental considerations in the thermal performance of panels. Structures equipped with the formulated panels consume less energy for thermal comfort, ultimately reducing fuel and energy consumption.

### 3.3. Specific Heat Capacity of Panel Specimens

**Figure 7:**
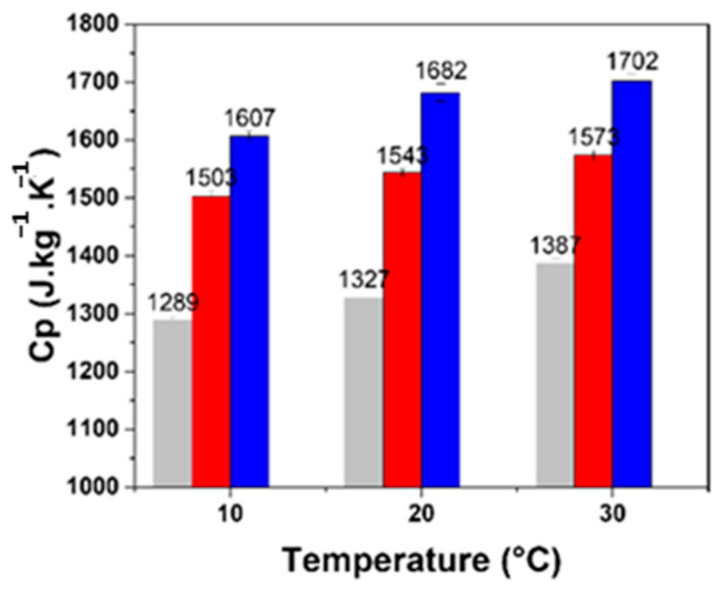
Specific heat capacity of specimens with different proportions of BSGs.

The specific heat capacity values for the formulated panels range from 1327 to 1682 J·kg^−1^·K^−1^ at 20 °C, as shown in Figure 7. Notably, at all measured temperatures, the specific heat capacity (Cp) of panels containing only Brewers’ Spent Grains as granular (BSG100) is significantly higher than that of the formulation with hemp shives (BSG0) or the mixed formulation (BSG50), as illustrated in Figure 6. This increase in specific heat capacity is attributed to the unique properties of BSGs, which exhibit low thermal conductivity, alongside a high specific heat capacity.

In the construction industry, materials with elevated calorific capacity are essential for enhancing energy efficiency. The specific thermal capacity is crucial for maintaining indoor comfort for building occupants. Walls constructed with materials having high thermal capacity can effectively regulate temperatures, keeping spaces cooler in summer without the need for additional energy inputs. Conversely, in winter, such materials can help retain heat within buildings for extended periods.

In conclusion, the results indicate that the formulated panels, particularly those incorporating BSGs, demonstrate improved thermal performance compared to formulations composed solely of hemp shives or a blend of the two materials.

### 3.4. Mechanical Strength of Panel Specimens

The compressive strength of building materials is a crucial factor that determines their suitability for construction applications. In this study, we evaluated the compressive strength of the three considered formulations: BSG0, BSG50, and BSG100. The results of the compressive strength tests are presented in Table 4.

As illustrated in Table 4, the formulation containing only hemp shives (BSG0) exhibits the highest compressive strength at 0.327 MPa. This superior strength can be attributed to the inherent properties of hemp shives, which are known for their fibrous structure that contributes to load-bearing capacity [29]. Conversely, the mixed formulation (BSG50) shows a reduced compressive strength of 0.162 MPa, while the formulation comprising entirely BSGs (BSG100) demonstrates the lowest compressive strength, at 0.098 MPa.

The decrease in compressive strength observed in the BSG50 and BSG100 formulations can be linked to the porous nature and lower density of BSGs, thus affecting the overall mechanical performance of the material. Studies indicate that materials with higher porosity typically exhibit lower compressive strength due to the increased voids that compromise the structural integrity [30].

Additionally, the use of organic materials, such as BSGs, can enhance the sustainability of building materials, although they may contribute to lower compressive strength compared to traditional materials [31]. It is crucial to balance the benefits of using recycled and biosourced materials with their mechanical performance to optimize their application in construction.

In conclusion, while the BSG0 formulation demonstrates the highest compressive strength, the BSG100 formulation highlights the trade-off between incorporating sustainable materials and achieving required mechanical properties. Further research is needed to explore the potential of enhancing the compressive strength of BSG-based formulations, possibly through the incorporation of additives or composite strategies.

## 4. Discussion

The results of this study demonstrate the hygrothermal and mechanical properties of building panels formulated with BSGs and hemp shives, providing insights into their moisture sorption behavior, specific heat capacity, and mechanical strength. The incorporation of BSG as an alternative material in building construction presents both advantages and trade-offs. In this section, the findings are discussed in comparison with the existing literature, focusing on the implications for building material performance and sustainability.

### 4.1. Moisture Sorption Behavior

The sorption isotherms for all formulations exhibited a Type II isotherm, characteristic of porous materials, and this finding is consistent with findings in the literature on bio-based materials such as hemp and recycled agricultural waste [32,33]. Notably, BSG100, despite its lower porosity and higher density, displayed a significant capacity for moisture absorption, which can be attributed to the hydrophilic nature of BSGs. This aligns with previous studies that reported high moisture affinity in materials with similar organic compositions [34]. The porosity of hemp shives in BSG0 contributed to higher mass differentials during moisture absorption, yet the ability to retain moisture was lower than that of BSG100. This trend of lower moisture retention with higher porosity has been documented in materials like hemp composites [35].

### 4.2. Thermal Conductivity

Thermal conductivity testing revealed that the panels made solely from hemp shives (BSG0) exhibited superior insulation properties, with a thermal conductivity of 0.067 W/(m·K), which is significantly lower than the 0.131 W/(m·K) of the BSG100 formulation. The observed thermal conductivity values for BSG0 are in line with previous studies on hemp-based materials, where hemp–lime and hemp–shive composites have shown thermal conductivity ranging from 0.05 to 0.08 W/(m·K), as shown in Table 5. Meanwhile, the higher thermal conductivity of BSG100 can be attributed to the denser structure of BSG, thus reducing its insulating potential. This result is expected, as an increased density tends to give a material with higher thermal conductivity. The chemical compositions of the BSG- and hemp shive-based composites play a significant role in determining their thermal and mechanical performance. Brewer’s Spent Grains (BSGs), as seen in the Materials section, are composed of hemicellulose (22–40%), cellulose (12–33%), protein (14.2–26.7%), and lignin (11.5–27.8%), which contribute to the material’s porosity and density, factors that are crucial in defining its thermal conductivity and mechanical strength. In contrast, hemp shives, which consist of cellulose (44–51.6%), hemicellulose (6.4–27%), and lignin (8.1–28%), provide excellent thermal insulation due to their fibrous structure and low density. The higher cellulose content in hemp shives, compared to BSGs, is likely a key contributor to the lower thermal conductivity of the BSG0 formulation (0.067 W/(m·K)), which falls within the range typically observed for hemp-based materials. Meanwhile, the BSG100 formulation, with a thermal conductivity of 0.131 W/(m·K), demonstrates higher conductivity due to the denser structure of BSG, which reduces its insulating potential. This aligns with the general principle that higher density materials tend to have higher thermal conductivity. The increased porosity of the BSG100 formulation, driven by the BSG content, also compromises its mechanical strength, as seen in the lower compressive strength values.

Thermal conductivity is a key criterion in evaluating insulation materials, and the values obtained for BSG formulations, especially BSG50 and BSG0, align with the ranges reported for commonly used materials. This demonstrates that BSG-based composites can deliver competitive performance while leveraging an abundant industrial by-product. Additionally, the comparison of thermal conductivity with other studies highlights consistency with renewable aggregates like hemp shives and flax, reinforcing the potential of BSG as an efficient, eco-friendly insulation material.

### 4.3. Specific Heat Capacity

The specific heat capacity of the panels varied from 1327 J·kg^−1^·K^−1^ to 1682 J·kg^−1^·K^−1^, with BSG100 displaying the highest values across all temperatures tested. This observation corresponds with findings from studies on bio-based materials, where a high calorific capacity was noted for materials such as wood waste [36]. The high specific heat capacity of BSG formulations allows for better thermal regulation, a desirable property for energy-efficient buildings.

**Table 5:** Comparison of mechanical and thermal properties within the literature.

Property	Formulation	Used Materials	Measured Value	Literature Range	Literature Materials	References
**Thermal Conductivity (W/(m·K))**	BSG0	Hemp shives	0.067	0.05–0.08	Wheat/hemp shives+starch/coconut husk/rice husk	[37,38,39,40]
	BSG50	50% BSG + 50% hemp	0.106	0.1–0.15	Hemp shives+flax/wood fibers/corn cob	[38,41,42]
	BSG100	Brewers’ Spent Grains	0.131			
**Compressive Strength (MPa)**	BSG0	Hemp shives	0.327	0.2–0.4	Rye/hemp shives/reed/beet pulp+ starch	[38,43,44,45]
	BSG50	50% BSG + 50% hemp	0.162	0.06–0.2	Wheat/wheat starch+wheat straw/lavender/corn cob	
	BSG100	Brewers’ Spent Grains	0.098			[37,38,46,47]

### 4.4. Mechanical Strength

The mechanical strength of the formulated panels, especially in terms of compressive strength, indicated that BSG0 exhibited the highest value (0.327 MPa), followed by BSG50 (0.162 MPa) and BSG100 (0.098 MPa). The decreased compressive strength observed with increasing BSG content can be attributed to the increased porosity of BSG, which compromises the structural integrity of the material. Similar trends have been reported in the literature for bio-based and porous materials, for which high porosity results in reduced mechanical performance, as shown in Table 5. However, the lower strength of BSG100 may be counterbalanced by its environmental benefits and thermal advantages.

Table 5 gives a comparison of the results presented in this paper with some other building materials used for insulation (values issued from the literature). Both thermal properties and mechanical properties are equivalent when considering the materials of this study or other existing materials. The materials chosen as examples and found in the literature (Table 5) are not necessarily sourced from wastes, and, in some cases, they are produced specifically to be incorporated in building materials. With our approach focused on sustainability and circular economy, the BSG can come not as a replacement but as an alternative in localities where this waste is abundant. Then, using BSG would reduce the stress on conventional resources destined for building materials. Although fungal development was not observed on the samples for the period studied, we recommend additional studies toward understanding the durability of materials containing BSGs.

### 4.5. Recommendations

Based on the findings of this study, several recommendations can be made for the application of BSG- and hemp-based materials in construction:

**Optimizing the blend of materials**: The use of a mixed formulation (BSG50) provides a balance between moisture sorption, thermal conductivity, and mechanical strength. Further optimization of the blend ratio, possibly combined with additives, could enhance the overall performance of the material.

**Focus on thermal performance for energy efficiency**: The high specific heat capacity of BSG100 makes it a suitable material for passive thermal regulation in buildings. Incorporating BSG into panel systems could reduce energy demands for heating and cooling, contributing to sustainability goals in the construction industry.

**Addressing fungal development possibilities**: The high moisture absorption capacity of BSGs can be beneficial for regulating humidity in indoor environments. However, strategies to prevent excessive moisture retention, such as surface treatments or incorporating moisture barriers [48], should be considered to avoid potential issues with mold growth or material degradation.

## 5. Conclusions

This study demonstrates the potential of BSGs and hemp shives, along with cornstarch, as sustainable building materials. The main conclusions are as follows:

- The moisture sorption analysis reveals that all formulations exhibit Type II isotherm behavior, with BSG displaying superior moisture absorption due to its hydrophilic nature. Thermal conductivity tests show that while the BSG formulation has higher thermal conductivity than hemp shives alone, it still contributes to energy efficiency through enhanced thermal regulation.
- Mechanical strength assessment indicates that the formulation with only hemp shives (BSG0) exhibits the highest compressive strength, while BSG formulations (BSG50 and BSG100) demonstrate lower strength due to the increased porosity of BSG aggregates. However, the environmental benefits of using BSGs, including waste reduction and improved thermal properties, could outweigh these limitations.

These findings underscore the importance of balancing sustainability with mechanical performance in building materials. By integrating BSGs into construction practices, the industry can advance toward a more circular economy. Future research should focus on optimizing material blends, enhancing mechanical properties, and exploring fungal development to maximize the performance of BSG-based building materials. This study lays the foundation for further exploration into sustainable construction practices that leverage abundant by-products, contributing to both environmental goals and resource efficiency in the building sector.

## Figures and Tables

**Table 1:** Physical properties of BSGs and hemp shives.

	Diameter (mm)	Density (kg·m^−3^)	Initial Water Content (%)
Hemp shives	1–5	105 ± 10	7.6
Brewers’ Spent Grains	1–3	640 ± 50	10.3

**Table 2:** Chemical composition of the cornstarch.

Component	Content
Amylose (%)	26.55
Amylopectin (%)	73.67
Crude fats (%)	5.43
Crude proteins (%)	9.45
Ash (%)	0.87
Phosphor (%)	0.07
Moisture content (%)	10.8
Density (kg/m^3^)	1416

**Table 3:** Chemical composition of tap water used for the preparation of gelatinized starch.

Element	Ca	Mg	Na	Cl	K	P	F	Zn	SO_4_^−^	NO_3_^−^	Fe	Cu	Mn
**Concentration (mg/L)**	95.3	6.8	12.5	18.1	3	0.7	0.2	0.2	37.6	43.4	<0.1	<0.1	<0.1

**Table 4:** Mean compressive strength results.

Formulations	Compressive Strength (MPa)
BSG0	0.327 ± 0.03
BSG50	0.162 ± 0.02
BSG100	0.098 ± 0.02

## Data Availability

The experimental and computational data presented in this paper are available from the corresponding author upon request.
